# Parallel Processing of Sobel Edge Detection on FPGA: Enhancing Real-Time Image Analysis

**DOI:** 10.3390/s25123649

**Published:** 2025-06-11

**Authors:** Sanmugasundaram Ravichandran, Hui-Kai Su, Wen-Kai Kuo, Dileepan Dhanasekaran, Manikandan Mahalingam, Jui-Pin Yang

**Affiliations:** 1 Department of Electro-Optics Engineering, National Formosa University, Yunlin County 632301, Taiwan; rsanmu88@gmail.com (S.R.); wkkuo@nfu.edu.tw (W.-K.K.); 2 Department of Electrical Engineering, Smart Machinery and Intelligent Manufacturing Research Center, National Formosa University, Yunlin County 632301, Taiwan; 3 Department of Electronics & Communication Engineering, Vel Tech Rangarajan Dr Sagunthala R&D Institute of Science and Technology, Chennai 600062, India; dileepanphd@gmail.com; 4 SRM Institute of Science and Technology, Tiruchirappalli 621105, India; maniece022@gmail.com; 5 College of Marine Resources and Engineering, National Penghu University of Science and Technology, Penghu County 880, Taiwan; juipinyang@gmail.com

**Keywords:** Sobel edge detection, real-time processing, FPGA technology, computer vision, Verilog, matrix arithmetic, image processing algorithms, fixed-point arithmetic, future research, technology integration

## Abstract

Detection of object boundaries and significant features within an image is one of the most important processes in image processing and computer vision, as it allows the identification of object boundaries and significant features within an image. In applications such as autonomous vehicles, surveillance systems, and medical imaging, real-time processing has become increasingly important, which requires hardware accelerators. In this paper, the improved Sobel edge detection algorithm was implemented using Verilog as an FPGA-based algorithm designed to perform real-time image processing under the Sobel edge detection algorithm for specially RGB images. The proposed design proposes an application of horizontal and vertical Sobel kernels in parallel in order to compute the gradient magnitudes for 1028 × 720 RGB images by taking the gradient magnitudes of 3 × 3 pixel windows. This work focuses on algorithmic complex reduction by using eight directional approaches, and parallel processing leads to reducing the architectural utilization.

## 1. Introduction

In recent years, there has been a significant increase in the demand for high-speed and effective image processing solutions, driven by the advancement of autonomous systems, surveillance, and industrial automation systems [1]. There is no doubt that edge detection is an integral component of computer vision, as it is essential for the delineation of object boundaries and the extraction of key features to be further analyzed. Due to the inherent limitations in computational speed and power efficiency in traditional software implementations of edge detection algorithms, such as the Sobel operator, many of the real-time processing requirements necessary for these applications cannot be met in a timely manner due to their inherent limitations [2]. Thus, the use of field-programmable gate arrays (FPGAs) in order to implement hardware acceleration has become a compelling solution for enhancing the speed and efficiency of processing, since they offer the capability to perform complex computations in parallel, thereby enhancing processing speed and efficiency [3].

The paper discusses the implementation of the Sobel edge detection algorithm on an FPGA using Verilog, focusing on the use of a modular design to take advantage of the parallel processing capabilities of the FPGA in order to produce a performance that is as near to real time as possible. In order to ensure that significant intensity changes are highlighted, the Sobel operator uses a gradient-based convolution method, which effectively highlights significant intensity changes, making it ideal for parallel use on FPGA platforms [4]. Our work focuses on designing a convolution module that computes both horizontal and vertical gradients simultaneously using separate kernels of the Sobel algorithm using the techniques described in this paper. This system is capable of processing high-resolution images in real time due to the parallel implementation of these convolutions, making it ideally suited to processing high-resolution images in real time due to its high throughput.

It has been demonstrated in this work [5] that real-time image processing solutions can be successfully deployed in resource-constrained environments, especially in applications that demand both high performance and low power consumption, such as embedded vision systems [6]. During the development of these systems, three key requirements need to be met: (1) Strict real-time performance in order to meet the demands of applications that are sensitive to latency. (2) High-quality edge detection to ensure accuracy in identifying features, as inaccuracies can significantly degrade the overall performance of the system. (3) Robustness across a range of conditions, including different lighting settings and noise levels [7]. There are many limitations associated with traditional processors and GPUs based on traditional hardware, and FPGA technology is able to overcome these challenges, as well as the high computational load and energy inefficiencies associated with image processing. As a result of this approach, which focuses on simplifying edge detection algorithms, including the Sobel operator, and optimizing their implementation on FPGAs, we hope to achieve enhanced processing speed and reduce complexity, particularly when it comes to applications like boundary detection and object recognition in dynamic settings [8].

A variety of edge detectors, including Sobel, Prewitt, Roberts, Laplacian, and Canny, have unique advantages and limitations in terms of their underlying methods for detecting intensity discontinuities [9,10,11]. Comparatively, the Roberts operator fails to capture finer edges, resulting in incomplete edge maps. As a result of this limitation, Roberts has not been able to succeed in applications requiring precise edge details, where its ability to rely on very localized pixel differences is a hindrance. Alternatively, the Laplacian operator, which is a second-order derivative method, is highly sensitive to noise, and it often produces double edges as a result, which complicates the interpretation of the edge direction and reduces its effectiveness in the detection of clean edges.

It is widely recognized that the Canny edge detector is one of the most robust methods for detecting edges. As a result of the multi-stage approach that includes Gaussian smoothing, gradient computation, and double thresholding, it achieves this result. By using this technique, Canny is capable of reducing noise, detecting both strong and weak edges, and providing an edge map that closely matches the original image structure in terms of its edge detection. In real-time or resource-constrained environments, such as FPGA-based implementations, simpler algorithms like Sobel offer significant advantages over the Canny operator due to their complexity and computational intensity. Using the Sobel operator, we are able to calculate the gradient of image intensity, which shows abrupt changes that indicate potential edging points. By enhancing edges while suppressing noise, the technique is effective in accentuating edges while reducing noise [9]. The study [12] discusses the reasons behind the use of the Sobel operator for edge detection, when more advanced methods such as the Canny and Laplacian Gaussian methods are available. In addition to its simplicity and effectiveness, the Sobel operator provides a good balance between differing and smoothing. A convolution method based on two 3 × 3 kernels is used to calculate gradient magnitude horizontally and vertically. Because of this, edges can be efficiently highlighted, and noise can be reduced to some extent at the same time. Compared to more complex algorithms such as the Canny detector, the Sobel algorithm reliably captures main edges that represent significant intensity changes in an image, despite the fact that it produces less detailed edge maps. It is because of this capability that Sobel is ideal for applications where a fast and computationally less intensive method of edge detection is required [13]. As a result of its ability to perform noise suppression with moderate efficiency without the computational overhead of more complex algorithms, it will continue to be important in image processing and computer vision in the future. In light of the challenges faced by traditional algorithms, Sobel edge detection with field-programmable gate arrays (FPGAs) offers a compelling alternative. There are several features of FPGAs that make them stand out against other types of processing devices; however, the Cyclone V FPGA has the advantage of being able to be reconfigured [14].

In order to keep up with technological advancements, reconfigurable field-programmable gate arrays (FPGAs) are becoming more and more popular [15]. The flexibility of the FPGA allows for customized detection algorithms to be implemented, optimizing resource utilization and allowing applications to be tailored to specific requirements. This technique can be extremely useful in situations where the processing demands will vary depending on the situation or if power consumption has to be managed carefully [16]. The conventional approach to image processing today often entails reading an entire image into a RAM-based framebuffer, leading to escalating memory requirements, especially with increasing image resolutions, as more information is read into it. Real-time processing becomes a bottleneck as a result of this, leading to alternatives being sought. There are several hardware-centric techniques designed for FPGA-based Sobel identification that focus on efficient processing of the kernels of 3 × 3 pixels for optimal performance [17]. An FPGA-based solution offers several advantages over software implementations in this regard, including parallel processing capabilities that facilitate the extraction and processing of image kernels without requiring storing the entire frame in memory, which is not feasible when working with high-volume video data. A significant advantage of this approach is to reduce the amount of memory required per edge detection system. While maintaining a high image quality, this technique highlights the potential of FPGA architectures for enhancing the efficiency of Sobel filters in real-time edge detection applications.

There is a focus in this paper on how FPGA technology, based on the Cyclone V FPGA board, can be utilized for real-time edge detection. There are a number of technical aspects to the Sobel filter that we will explore in the following sections, including its role and pin assignments. This project uses Verilog to implement a Sobel edge detection algorithm on an FPGA and demonstrates the advantages of hardware acceleration in edge detection. In the first part of this article, an overview of edge detection algorithms is provided, with a focus on Sobel filtering since it is a simple, fast way to highlight changes in intensity due to its simplicity and efficiency. There is considerable evidence that FPGA implementations are more efficient than traditional software-based approaches, especially when it comes to Sobel edge detection, which combines simplicity and accuracy with the benefits of hardware acceleration over traditional software approaches. A comparison of these two applications illustrates the transformative ability of FPGA acceleration in the field of image processing. In this paper, we describe the key features of the FPGA that are relevant to the edge detection application. These include the FPGA family, device model, and the top-level entity that makes up the application. The importance of precise pin assignments is emphasized, with validation processes being utilized to ensure that the pins are connected and are working properly. Furthermore, the section discusses the considerations that were made during the implementation of the system, emphasizing the ways in which these design choices support robust performance of the system. As part of Verilog implementation, a signal pipeline is an important component that plays an integral role in handling the continuous stream of pixel data necessary for the edge detection process. We have put a lot of emphasis on the mechanisms for maintaining efficient image data processing and ensuring quality edge detection results by emphasizing synchronization and delay control mechanisms. Even though the detailed Verilog code is not included for reasons such as redundancy, this paper provides an in-depth examination of the pipeline’s key components, showing how they work together to form the overall FPGA-based implementation. In addition, the Cyclone V Starter Kit includes a detailed description of the pinout configuration of the FPGA board as well as a Verilog module, which is used as the foundation design file for the Cyclone V. In this comprehensive exploration, we provide insight into the intricacies of a FPGA-based edge detection system, which can serve as an excellent platform for understanding its design, implementation, and application to a variety of real-time image processing tasks.

This paper presents an FPGA-based implementation and evaluation of an improved Sobel edge detection algorithm. Initially, the traditional Sobel method is reviewed, followed by the introduction of an enhanced version designed to improve edge accuracy and reduce noise sensitivity. The system architecture includes key components such as the image control module, convolution module, output buffer, and synchronization logic, all optimized for real-time processing. The FPGA implementation is modular and efficiently handles image data flow. Results show that the improved Sobel algorithm outperforms conventional methods in both detection accuracy and hardware resource utilization, confirming its suitability for embedded image processing applications.

## 2. Materials and Methods

### 2.1. Trditional Sobel Filter Algorithm

The Sobel filter, a cornerstone in edge detection algorithms, forms the basis of our edge detection system. This section provides an in-depth look into the workings of the Sobel filter, detailing its horizontal and vertical processing matrices operating on a 3 × 3 image region. The combination of these matrices, involving square and square root operations, yields a strength value (G) representing the presence of edges in an image. The Sobel filter operates by processing a 3 × 3 image region for both horizontal and vertical components. These components are squared, and the square root is taken, resulting in a value ‘G’, representing the edge strength in the image. The algorithm calculates edge strength (‘G’) for vertical edges, providing insights into the algorithm’s behavior. Similar calculations for diagonal or sloped edges are encouraged for further understanding. The algorithm’s output is visualized by creating images where edges are visible as black, providing a tangible result for evaluation. The Sobel filter is commonly employed for edge detection using convolution kernels. For a pixel (x, y) in the image:

Horizontal Gradient ($G_{x}$):

$$\left|\begin{array}{ccc} P_{00} & P_{01} & P_{02}\\ P_{10} & P_{11} & P_{12}\\ P_{20} & P_{21} & P_{22} \end{array}\right|$$

(1) $$G_{x}=(P_{02}+2P_{12}+P_{22})-(P_{00}+2P_{10}+P_{20})$$

Vertical Gradient ($G_{y}$):

$$\left|\begin{array}{ccc} P_{00} & P_{01} & P_{02}\\ P_{10} & P_{11} & P_{12}\\ P_{20} & P_{21} & P_{22} \end{array}\right|$$

(2) $$G_{y}=(P_{20}+2P_{21}+P_{22})-(P_{00}+2P_{01}+P_{02})$$

Gradient Magnitude (G):

(3) $$\left|G\right|=\sqrt{G_{x}^{2}+G_{y}^{2}}$$

Approximate Magnitude (G):

(4) $$\left|G\right|=\left|G_{x}\right|+\left|G_{y}\right|$$

Angle of orientation of the edge (q):

(5) $$q=\arctan\left(\frac{G_{y}}{G_{x}}\right)$$

where

$I_{x}{,}_{y}$ is the intensity of the pixel at coordinates (x, y);

$G_{x}$ and $G_{y}$ are the horizontal and vertical gradients, respectively;

G is the magnitude of the gradient.

The convolution matrices employed in this design are as follows:

Kernel1 (Horizontal Gradient—$G_{x}$):

10−120−2−10−1

Kernel2 (Vertical Gradient—$G_{y}$):

121000−1−2−1

These kernels are applied to the pixel data within the defined 3 × 3 window. The algorithm processes the image by multiplying each pixel’s intensity by the corresponding kernel value, with the results stored in intermediate registers for subsequent operations. The horizontal ($G_{x}$) and vertical ($G_{y}$) gradients are calculated by summing these products, providing the gradient components that signify changes in image intensity. To further enhance the accuracy of edge detection, the code squares the computed horizontal and vertical gradients separately. The resulting values are then summed to approximate gradient magnitude, which indicates the edge strength. However, instead of performing a square root operation to derive the actual gradient magnitude, the design opts for a more resource-efficient approach by applying a threshold to this summed value. This threshold mechanism simplifies computation, resulting in a binary output that distinctly marks the presence or absence of an edge. The operational flow of the module is governed by the clock signal, ensuring that the pixel data is processed sequentially and in sync with the system’s timing constraints. The final output is a binary image where edges are represented by high-intensity pixels (8’hff), and non-edge regions are marked with low intensity (8’h00). This binary edge map is instrumental in applications such as lane detection, where the identification of sharp intensity transitions is critical.

### 2.2. Improved Sobel Algorithm

To address the limitations of traditional Sobel edge detection—namely, reduced edge accuracy and high hardware resource consumption—an improved algorithm [18,19] has been developed for implementation on FPGA using an 8-bit architecture. This enhanced Sobel method expands the detection scope from the conventional two directions (horizontal and vertical) to eight directional gradients: 0°, 45°, 90°, 135°, 180°, 225°, 270°, and 315°. These directions allow for more precise detection of edges that occur at diagonal and oblique orientations, significantly improving image boundary localization.

The original Sobel operator relies on convolution with pixel-weighted masks and calculates the gradient magnitudes in the x and y directions using

(6) $$G_{x}=f_{1}-f_{2},G_{y}=f_{3}-f_{4}$$

where

(7) $$f_{1}=p_{3}+2p_{6}+p_{9}$$

(8) $$f_{2}=p_{1}+2p_{4}+p_{7}$$

(9) $$f_{3}=p_{1}+2p_{2}+p_{3}$$

(10) $$f_{4}=p_{7}+2p_{8}+p_{9}$$

The overall gradient magnitude G is then given by Equation (11)

(11) $$\left|G\right|=\left|G_{x}\right|+\left|G_{y}\right|$$

However, for efficient FPGA implementation, especially under an 8-bit constraint, the formulae are simplified by right-shifting operations that replace multiplication/division. Specifically,

(12) $$G_{x}=\frac{1}{4}(f_{1}-f_{2}),G_{y}=\frac{1}{4}(f_{3}-f_{4})$$

For eight directions, a total of eight such gradient values are computed using rotated Sobel kernels. Due to the symmetry of these kernels and the use of absolute gradient values, only four directions (0°, 45°, 90°, and 135°) are required for calculation, with the other four being their mirror counterparts. The simplified total gradient in Equation (13):

(13) $$G=G_{0}+G_{45}+G_{90}+G_{135}$$

where

$$G_{0{}^{\circ}}=\left|\begin{array}{ccccc} 0 & 0 & 0 & 0 & 0\\ -1 & -2 & -4 & -2 & -1\\ 0 & 0 & 0 & 0 & 0\\ 1 & 2 & 4 & 2 & 1\\ 0 & 0 & 0 & 0 & 0 \end{array}\right|$$

$$G_{45{}^{\circ}}=\left|\begin{array}{ccccc} 0 & 0 & 0 & -1 & 0\\ 0 & -2 & -4 & 0 & 1\\ 0 & -4 & 0 & 4 & 0\\ -1 & 0 & 4 & 2 & 0\\ 0 & 1 & 0 & 0 & 0 \end{array}\right|$$

$$G_{90{}^{\circ}}=\left|\begin{array}{ccccc} 0 & -1 & 0 & 1 & 0\\ 0 & -2 & 0 & 2 & 0\\ 0 & -4 & 0 & 4 & 0\\ 0 & -2 & 0 & 2 & 0\\ 0 & -1 & 0 & 1 & 0 \end{array}\right|$$

$$G_{135{}^{\circ}}=\left|\begin{array}{ccccc} 0 & 1 & 0 & 0 & 0\\ -1 & 0 & 4 & 2 & 0\\ 0 & -4 & 0 & 4 & 0\\ 0 & -2 & -4 & 0 & 1\\ 0 & 0 & 0 & -1 & 0 \end{array}\right|$$

Further optimization involves reducing repeated calculations across the 16 possible sign combinations of the gradient components. These combinations are reduced to 8 cases by using absolute value symmetry, and common terms across these cases are extracted into intermediate sums, such as the following:

The final gradient is obtained by selecting the maximum among the simplified combinations:

(14) $$G=\max(f_{13},f_{14},f_{15},f_{16})$$

This calculated gradient is then evaluated using the threshold logic mentioned above in Equation (14).

**Figure 1:**
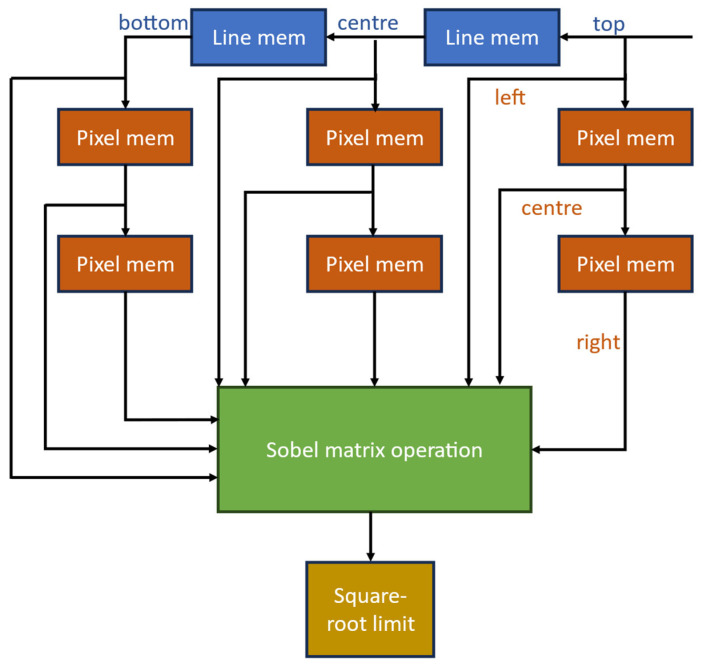
Improved Sobel operation with 8 directions.

This modified Sobel algorithm significantly improves edge detection precision while maintaining a lightweight design. The use of 8-bit architecture and symmetric kernel reuse helps in reducing hardware usage by over 13.9% in registers and 20.7% in logic blocks, compared to prior designs. Figure 1 illustrates the improved Sobel operation with 8 directions.

To detect horizontal edges, the Sobel algorithm uses a kernel that emphasizes differences in the vertical direction (i.e., changes in pixel values from top to bottom). This is referred to as the Sobel Y kernel or vertical edge detection kernel, and is represented as

$$G_{y}=\left[\begin{array}{ccc} P1 & P2 & P3\\ 0 & 0 & 0\\ P4 & P5 & P6 \end{array}\right]$$

When this kernel is convolved with the 3 × 3 neighborhood of a pixel, it computes the difference between the sum of the lower row of pixels and the sum of the upper row. The central row is multiplied by zero, indicating no contribution from pixels along the same row.

For detecting vertical edges, the algorithm uses the Sobel X kernel, which focuses on differences across the horizontal direction (i.e., left to right changes). The kernel used is

$$G_{x}=\left[\begin{array}{ccc} P1 & 0 & P4\\ P2 & 0 & P5\\ P3 & 0 & P6 \end{array}\right]$$

This matrix calculates the difference between the right-hand side pixels and the left-hand side pixels in the 3 × 3 neighborhood. The center column, multiplied by zero, contributes nothing to the result.

For a given pixel, the image is convolved with both $G_{x}$ and $G_{y}$ to compute the gradients in the x and y directions. The resulting gradient magnitude at the pixel location is approximated as Equation (3).

However, since square roots are computationally expensive, especially in hardware implementations, this expression is commonly approximated as Equation (11).

**Figure 2:**
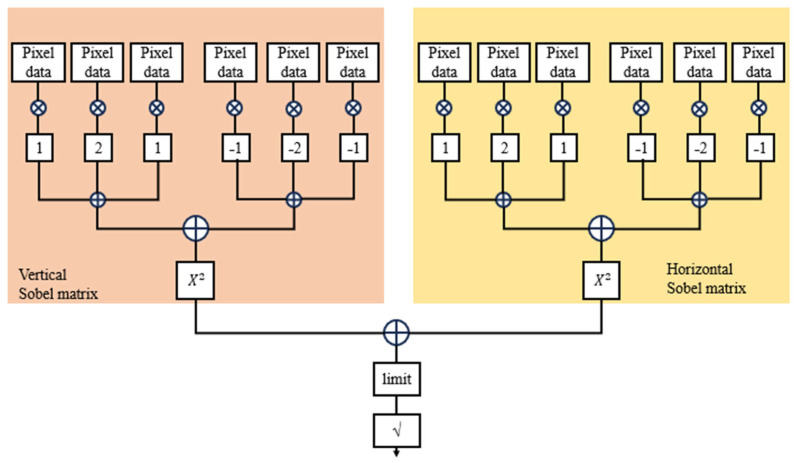
Horizontal G_x_ and vertical G_y_ calculator architecture.

This simplification provides a reasonably accurate estimate of edge strength with significantly less computational effort, making it suitable for FPGA or real-time image processing systems. Figure 2 represents horizontal Gx and vertical Gy calculator architecture.

### 2.3. System Architecture Overview for FPGA-Based Sobel Edge Detection

To begin, the test image is input using MATLAB R2024b, which is typically configured as an 8-bit grayscale image. The required BRAM memory size and the number of BRAMs needed are determined using the following formulas:

(15) $$\mathrm{Required}\;\mathrm{BRAM}\;\mathrm{memory}\;\mathrm{size}\left(Kbits\right)=\frac{\left(\mathrm{Image}\;\mathrm{dimensions}\;\times 8\right)}{1024}$$

(16) $$\mathrm{No}\;\mathrm{of}\;\mathrm{BRAMs}\;\mathrm{used}=\frac{\mathrm{Required}\;\mathrm{BRAM}\;\mathrm{memory}\;\mathrm{size}\;(\mathrm{Kbits})}{\mathrm{BRAM}\;\mathrm{size}\;(\mathrm{Kbits}\;\mathrm{per}\;\mathrm{module})}$$

**Figure 3:**
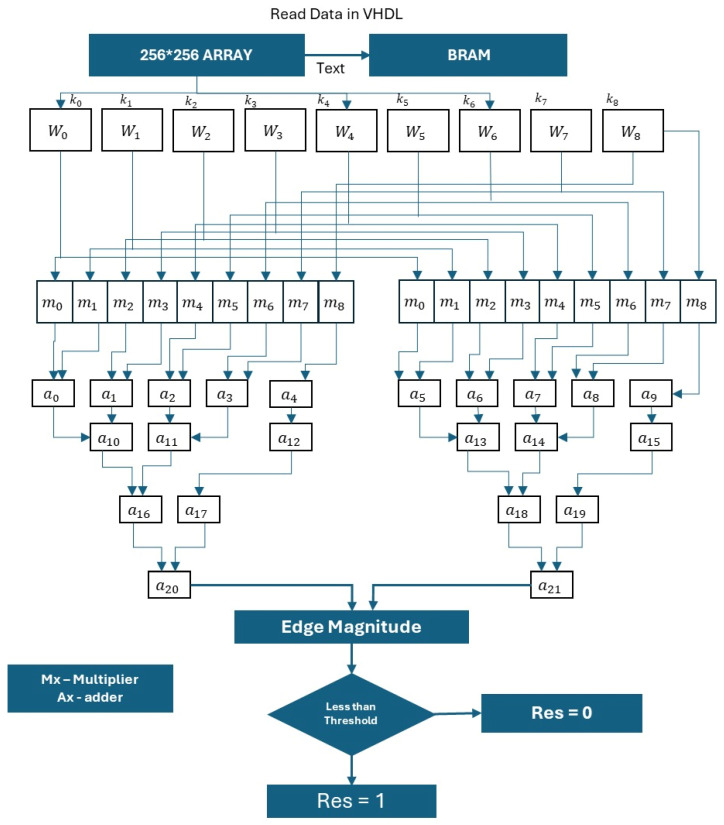
Improved Sobel edge detection architecture 8-bit parallel processing.
- Data Ready Signal: The o_data_ready signal is derived from the axis_prog_full signal and indicates to the upstream system whether the buffer can accept more data.

For a 512 × 512 image, this equates to around 2 Mbits, making efficient use of the FPGA’s block RAM resources. This setup is crucial for the subsequent edge detection process using the Sobel operator, which performs convolution using horizontal and vertical kernels to calculate gradient magnitudes. The processing is further enhanced by specialized IP cores, such as a Square Root Generator, to compute the final edge strength. This approach harnesses the FPGA’s ability to perform parallel processing and reconfigurability, enabling effective real-time image processing and edge detection. The manipulation of image data begins with importing test images, typically 8-bit grayscale, using MATLAB R2024b or another compatible tool. The images are resized and converted into hexadecimal values, which are then stored in a .mif or .hex file format compatible with FPGA block RAM initialization. The Cyclone V FPGA’s block RAM modules facilitate fast data access and processing, essential for the Sobel edge detection algorithm implemented in this project. The Main Module and 3 × 3 Block RAM within the FPGA coordinate data flow and buffering, ensuring continuous processing without delays. Central to this architecture is the Sobel Core, which employs dedicated horizontal ($G_{x}$) and vertical ($G_{y}$) convolution matrices to calculate the gradient magnitudes, effectively detecting edges by analyzing intensity changes within the pixel neighborhoods. The processed edge data is subsequently sent back to the PC, where the Output to Image Conversion module visualizes the edges, making the results readily interpretable for further analysis or integration into higher-level image processing tasks. The system architecture of the imageProcessTop module is designed to perform real-time image processing using field-programmable gate arrays (FPGAs), specifically implementing edge detection using the Sobel filter. This design takes advantage of the parallel processing capabilities and reconfigurable nature of FPGAs to achieve high-performance image processing. The architecture comprises several interconnected modules—image control (IC), convolution (Conv), and output buffer (OB)—which collectively form a processing pipeline that handles image data efficiently. Figure 3 illustrates improved Sobel edge detection architecture 8-bit parallel processing.

#### 2.3.1. Module Overview

(A) Top-Level Module (imageProcessTop):

The top-level module imageProcessTop acts as the central control unit that orchestrates data flow among the internal processing modules and interfaces with external components. It handles synchronization, data validation, and flow control, ensuring that each module operates in harmony to achieve the desired image processing outcomes.

1. Inputs:
  - axi_clk: The clock input that synchronizes all operations across the modules.
  - axi_reset_n: An active-low reset signal used to initialize the system and ensure all components start in a known state.
  - i_data_valid: A signal indicating that valid image data is available for processing.
  - i_data: An 8-bit input representing the grayscale image data stream.
  - i_data_ready: A signal from the downstream system indicating readiness to accept data.
2. Outputs:
  - o_data_ready: Indicates the readiness of the system to receive new data.
  - o_data_valid: Signifies that the processed data is valid and ready for transfer.
  - o_data: The 8-bit output data stream representing the processed image.
  - o_intr: An interrupt signal generated by the Image Control module for signaling system events or errors.

#### 2.3.2. Image Control Module (IC)

The image control module (IC) is responsible for receiving the raw input data, validating it, and formatting it for the convolution process. This module captures an 8-bit grayscale image data stream and manages the necessary synchronization and data alignment required for downstream processing.

Functionality:

- Data Handling: The module receives pixel data (i_pixel_data) and its corresponding validity signal (i_pixel_data_valid). It organizes this data into a 72-bit output (o_pixel_data), which represents a 3 × 3 block of pixels formatted for convolution.
- Reset Control: A reset signal (i_rst) derived from the top-level reset input (axi_reset_n) initializes the module. This reset ensures that all data paths and control signals are in their default states at startup.
- Interrupt Generation: The module can generate an interrupt signal (o_intr) to indicate specific events such as completion of a processing batch or detection of an error condition.

### 2.4. Convolution Module (conv)

The convolution module (Conv) implements the Sobel filter to perform edge detection on the input image. It processes the 3 × 3 pixel blocks received from the image control module using two sets of convolution kernels to compute the gradient in horizontal ($G_{x}$) and vertical ($G_{y}$) directions.

Functionality:

- Sobel Operation: The module uses two sets of Sobel kernels: one for horizontal gradients (kernel1) and another for vertical gradients (kernel2). For each 3 × 3 pixel block, the module computes the dot product of the pixel values with the kernel values, producing the gradients $G_{x}$ and $G_{y}$.
- Gradient Calculation: The gradients are squared and summed (convolved_data_int = convolved_data_int1 + convolved_data_int2) to produce the gradient magnitude, which represents the strength of edges at each pixel location.
- Thresholding: A threshold operation is applied to the gradient magnitude. If the magnitude exceeds a predefined threshold (4000 in this case), the pixel is marked as an edge (o_convolved_data = 8’hff); otherwise, it is not (o_convolved_data = 8’h00).

### 2.5. Output Buffer Module (OB or outputBuffer)

The output buffer module (OB) manages the final stage of data transfer, interfacing the processed data with external systems. It temporarily stores the convolved image data and ensures that data is only sent out when the receiving system is ready.

Functionality:

- AXI Streaming Interface: The module uses AXI streaming protocols, with signals like s_axis_tvalid, s_axis_tready, and m_axis_tvalid managing the flow of data into and out of the buffer. These signals ensure proper handshaking between the FPGA and the external receiver, preventing data loss or overflow.
- Data Flow Control: A programmable full signal (axis_prog_full) indicates when the buffer is reaching capacity. This signal is used to throttle incoming data, ensuring that the buffer does not overflow, which could cause data corruption.

### 2.6. System Synchronization and Data Flow

**Figure 4:**
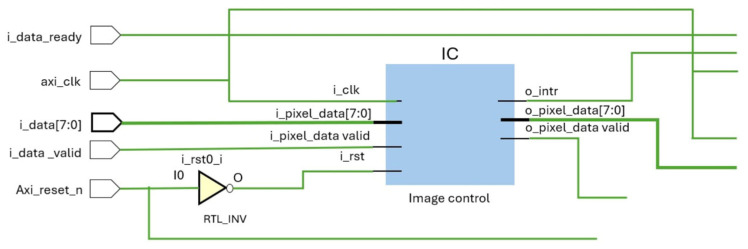
Image Control module (IC) integration.

**Figure 5:**
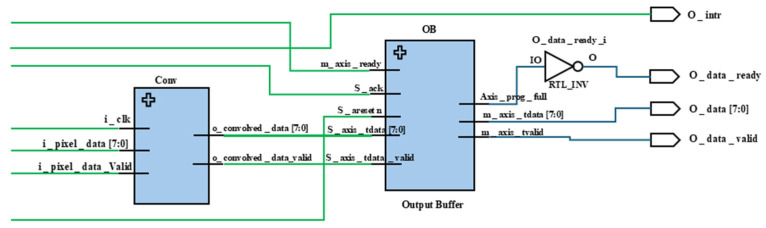
Output buffer and convolution module (Conv) integration.

- Clock Domain Synchronization: The entire system is synchronized using a single clock domain (axi_clk). This ensures that all modules process data in lockstep, avoiding timing mismatches that could lead to data corruption.
- Data Validation: Throughout the system, data validity signals (i_data_valid, o_convolved_data_valid, etc.) are used to manage data flow, ensuring that only valid data is processed and transferred between modules.
- Flow Control and Interrupt Handling: The architecture includes comprehensive flow control mechanisms, such as the use of the o_data_ready signal to manage the input data rate and the o_intr signal to handle interrupts for system-level events. Figure 4 illustrates image control module (IC) integration and Figure 5 represents output buffer and convolution module (Conv) integration.

**Figure 6:**
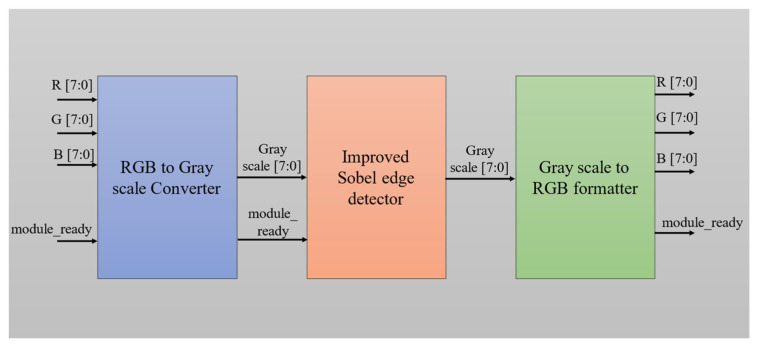
Improved Sobel Edge Detector for RGB format.

The above Figure 6. represents a hardware pipeline architecture for real-time edge detection using an improved Sobel algorithm. This system is composed of three main processing blocks: the RGB to Grayscale Converter [18,20], the Improved Sobel Edge Detector, and the Grayscale to RGB Formatter, each operating on 8-bit pixel data and controlled using a module_ready signal to synchronize the data flow across stages.

The process begins with the RGB to Grayscale Converter, which receives three 8-bit inputs corresponding to the red (R), green (G), and blue (B) color channels of a pixel. Using a standard luminance-preserving formula—typically a weighted sum such as Gray = 0.299 × R + 0.587 × G + 0.114 × B—the converter reduces the colored input into a single 8-bit grayscale value. This simplifies the subsequent edge detection process by eliminating the need to process each color channel independently.

The grayscale output is then passed to the Improved Sobel Edge Detector. This module enhances traditional Sobel processing by applying convolution kernels in eight different directions: 0°, 45°, 90°, 135°, 180°, 225°, 270°, and 315°, thereby capturing edge features more accurately in all orientations. To maintain hardware efficiency, the design uses an 8-bit architecture and shift-based arithmetic rather than multipliers. The output of this module is a refined grayscale image where high-intensity pixel values represent detected edges.

Finally, the Grayscale to RGB Formatter takes the edge-highlighted grayscale output and replicates its value across all three-color channels—R, G, and B—thus converting it back into an RGB format. This step ensures that the resulting image is compatible with standard display or image storage systems, even though the content remains visually grayscale. The module_ready signal at each stage manages synchronization, ensuring that data is correctly handed off between modules without timing conflicts. Overall, this architecture supports a high-performance and resource-efficient solution for real-time edge detection in FPGA-based image processing applications.

## 3. Results

**Figure 7:**
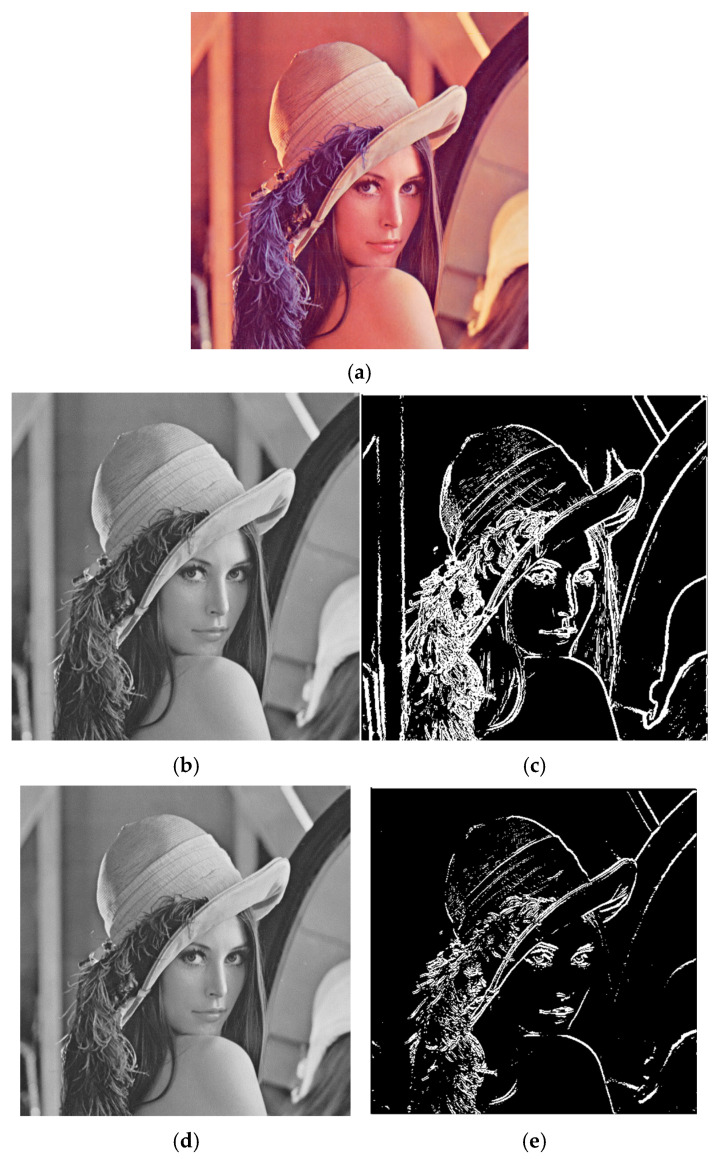
Implementation of the Sobel filter on the Lena_Gray image [19].

The results demonstrate that when both horizontal and vertical gradients are applied simultaneously, the system effectively captures multi-directional edges, providing a comprehensive edge map that enhances feature detection in complex images. This combined approach leverages the full capability of the FPGA’s parallel processing, utilizing the Sobel filter’s convolution operations on both axes to detect a wide range of edge orientations. Figure 7a represents the original image, and Figure 7b represents the original grayscale image, which serves as the input for the edge detection algorithms. This image is crucial as it provides the baseline against which the effectiveness of the edge detection methods is assessed. In our analysis, we used a 512 × 512 pixel grayscale image to maintain uniformity and ease of processing within the FPGA-based Sobel filter framework. Figure 7c illustrates the result of applying the Sobel filter with both horizontal ($G_{x}$) and vertical ($G_{y}$) gradients. This combined approach effectively highlights edges in all directions, making it an ideal technique for general edge detection tasks. The image clearly shows enhanced edges corresponding to significant changes in intensity, demonstrating the Sobel filter’s ability to capture both vertical and horizontal features within the original image. Figure 7d depicts the output when the Sobel filter is applied using only the horizontal gradient ($G_{x}$). This method focuses on detecting vertical edges within the image, as it emphasizes changes in pixel intensity along the horizontal axis. The resulting image highlights structures such as walls, posts, or any other features that align vertically in the original image, providing a clear visualization of vertical boundaries. Figure 7e shows the result of applying the Sobel filter using only the vertical gradient ($G_{y}$). This approach accentuates horizontal edges by detecting variations in pixel intensity along the vertical axis. The resulting image emphasizes horizontal features such as road lines, horizons, or other horizontal structures, effectively isolating these elements from the rest of the image.

Resource utilization analysis is a critical aspect of FPGA design evaluation (Table 1), offering insights into how efficiently the available hardware resources are used to implement a given system. In FPGA development, resources such as Look-Up Tables (LUTs), Slice Registers, Block RAMs, and DSPs are finite and must be allocated judiciously to meet design specifications while optimizing performance and minimizing costs. 

The resource utilization analysis indicates a well-optimized design with balanced use of logic and memory resources. While the top-level and control modules are logic-heavy, the use of Block RAM remains minimal, providing ample opportunity for further enhancement or expansion of memory-intensive operations. The low utilization of DSPs also suggests potential for integrating more complex algorithms without significant redesign, aligning the current resource use with the goals of performance and scalability in FPGA-based image processing applications.

In Table 2, comparative analysis [21] of the architecture, data format, speed, hardware resources, frame resolution, and platform used in each study are compared. The architecture used in the current work is parallelized edge detection, which is designed for efficient processing. In contrast, the other works employ architectures like overlapping pipeline, inverse Hough transform, and dual-stage landmark detection. All systems use the floating point data format. The speed of the algorithm varies significantly, with this work achieving the fastest processing time of 0.0033 s, which is considerably faster than Khongprason’s 0.0076 s, Hajjouji’s 0.0147 s, and Malmir’s 0.04 s. Regarding hardware resources, the current method uses 1938 LUTs (Look-Up Tables), which is significantly lower than Khongprason’s 91,050 LUTs but higher than Malmir’s 1996 LUTs. The frame resolution for this work is 1028 × 720, compared to 480 × 270 for Khongprason, 640 × 480 for Hajjouji, and 1280 × 720 for Malmir. The platforms used for the implementations are diverse: DE1-SoC for this work, Zynq-7000 APSoC for Khongprason, Virtes-5 for Hajjouji, and KC705 evaluation board for Malmir.

Table 3 compares the area and resource consumption for the different edge detection techniques. The table splits the area into combinational and non-combinational areas, along with the total design area. For the current work, the combinational area is 187.452 units, and the non-combinational area is 178.623 units, resulting in a total design area of 366.075 units. This is much smaller than Khongprason’s 630.338 units and Malmir’s 898.308 units, but slightly larger than Hajjouji’s 295.569 units for the combinational area. The gate count indicates the total number of logic gates used, with this work using 241.379 gates, which is fewer than Khongprason’s 413.607 gates and Malmir’s 589.440 gates, but more than Hajjouji’s 257.193 gates. Finally, the RAM size required by this work is 5.12 kB, which is the smallest compared to 7.68 kB for Khongprason and 45 kB for Malmir.

Table 4 presents a quantitative comparison of various edge detection operators using four objective evaluation metrics: mean squared error (MSE), normalized cross-correlation (NK), average difference (AD), and normalized absolute error (NAE). These metrics help assess the fidelity and precision of detected edges with respect to the original image. Among the evaluated methods, the improved Sobel operator significantly outperformed traditional methods in terms of NK, AD, and NAE. It achieved the lowest NAE (0.1159) and the least AD (−0.0509), indicating highly accurate and minimally deviated edge maps. While the MSE for improved Sobel was higher than the others (0.3372), this is acceptable as MSE can be sensitive to the sparse pixel changes typical in edge maps. Overall, the improved Sobel operator offers superior detection accuracy, making it ideal for applications requiring fine edge details and structural clarity.

Table 5 provides a comparative overview [25] of FPGA slice logic utilization for each edge detection operator. Key hardware parameters such as the number of flip-flops, Slice Look-Up Tables (LUTs), logic LUTs, and total occupied slices were analyzed to determine implementation efficiency. The improved Sobel operator demonstrated a well-balanced hardware footprint, using fewer flip-flops (254) than both Prewitt (339) and Sobel (343), and moderately optimized logic usage (410) compared to others. Although its LUT usage was slightly higher (1938), this reflects the added logic complexity required to enhance edge precision. Importantly, the total number of occupied slices (473) was less than that of Prewitt and Sobel, indicating improved layout efficiency. These results confirm that the improved Sobel is not only more accurate but also more hardware-efficient, making it suitable for real-time embedded or resource-constrained FPGA environments compared with other operators.

These comparisons highlight how the proposed method achieves high speed and efficiency in edge detection while maintaining low hardware resource consumption, making it a competitive solution in terms of both performance and resource utilization compared to existing methods.

## 4. Conclusions

This paper presents an efficient FPGA-based implementation of the Sobel edge detection algorithm, demonstrating significant improvements in processing speed and resource utilization over traditional software-based approaches. By leveraging the parallel processing capabilities of FPGAs, the implementation effectively handles high-resolution images in real time, meeting the stringent performance requirements of modern image processing applications, such as autonomous systems and surveillance. The design utilizes separate horizontal and vertical convolution kernels to compute gradient magnitudes, highlighting the adaptability of FPGAs for modular and scalable image processing tasks. The analysis of resource utilization reveals an optimal balance between LUTs, registers, and Block RAM, ensuring that the system can be scaled or adapted for more complex algorithms without substantial redesign. Furthermore, the low DSP usage indicates potential for incorporating more sophisticated image processing techniques within the current hardware constraints. In summary, the FPGA-based Sobel edge detection system not only meets the real-time processing needs but also offers flexibility for future enhancements and scalability. This work underscores the potential of FPGAs in addressing the limitations of traditional image processing methods, paving the way for more advanced and efficient implementations in various real-world applications. Future work may explore the integration of additional edge detection algorithms, like the Canny edge detector, to further enhance the robustness and accuracy of the system in diverse operational environments.

## Figures and Tables

**Table 1:** Resource utilization analysis.

Name	Slice LUTs	Slice Register	Block Ram Tile	DSPs
Image process	1938	254	0.5	2
conv (conv)	114	85	0	2
IC	1773	109	0	0
OB	50	60	0.5	0

**Table 2:** Comparative analysis.

	This Work	Khongprason [22]	Hajjouji [23]	Malmir [24]
Architecture	Parallelized edge detection	Overlapping pipeline	Inverse Hough transform	Dual stage landmark detection
Data format	Floating point	Floating point	Floating point	Floating point
Speed in Sec	0.0033	0.0076	0.0147	0.04
Hardware resources (LUT)	1938	91,050	1996	38,220
Frame resolution	1028 × 720	480 × 270	640 × 480	1280 × 720
Platform	DE1-SoC (Cyclone V)	Zynq-7000 APSoC	Virtes-5	KC705 evaluation board

**Table 3:** Different edge detection techniques.

Area	Improved Sobel Edge Detection	Canny Edge Detection	Hough Transform
Combinational area	187.452	295.569	453.392
Non-combinational area	178.623	257.193	333.436
Design area	366.075	630.338	898.308
Gate count	241.379	413.607	589.440
RAM Size	5.12 KB	7.68 KB	45 KB

**Table 4:** Comparative analysis of various edge detection operators.

Operators	MSE	NK	AD	NAE
Zero-cross	0.1458	0.4516	−0.0725	2.1819
Prewitt	0.1416	0.4307	−0.123	8.7013
Roberts	0.1383	0.5342	−0.1253	9.8979
Sobel	0.1415	0.4301	−0.1236	8.9929
Log	0.1458	0.4516	−0.0725	2.1819
Improved Sobel	0.3372	0.1159	−0.0509	0.1159

**Table 5:** Comparative overview of FPGA slice logic utilization.

Slice Logic Utilization	Robert	Prewitt	Sobel	Improved Sobel
Number used as flip-flops	219	339	343	254
Number of Slice LUTs	1598	1727	1728	1938
Number used as logic	322	450	450	410
Number of occupied slices	462	490	495	473

## Data Availability

No new data were created or analyzed in this study. Data sharing is not applicable to this article.
